# Pharmacologic rescue of hyperammonemia-induced toxicity in zebrafish by inhibition of ornithine aminotransferase

**DOI:** 10.1371/journal.pone.0203707

**Published:** 2018-09-10

**Authors:** Matthias Zielonka, Maximilian Breuer, Jürgen Günther Okun, Matthias Carl, Georg Friedrich Hoffmann, Stefan Kölker

**Affiliations:** 1 University Hospital Heidelberg, Center for Child and Adolescent Medicine, Division for Pediatric Neurology and Metabolic Medicine, Heidelberg, Germany; 2 Heidelberg Research Center for Molecular Medicine (HRCMM), Heidelberg, Germany; 3 Heidelberg University, Medical Faculty Mannheim, Department of Cell and Molecular Biology, Mannheim, Germany; 4 University of Trento, Center for Integrative Biology (CIBIO), Laboratory of Translational Neurogenetics, Trento, Italy; National Center for Toxicological Research, UNITED STATES

## Abstract

Hyperammonemia is the common biochemical hallmark of urea cycle disorders, activating neurotoxic pathways. If untreated, affected individuals have a high risk of irreversible brain damage and mortality. Here we show that acute hyperammonemia strongly enhances transamination-dependent formation of osmolytic glutamine and excitatory glutamate, thereby inducing neurotoxicity and death in ammoniotelic zebrafish larvae via synergistically acting overactivation of NMDA receptors and bioenergetic impairment induced by depletion of 2-oxoglutarate. Intriguingly, specific and irreversible inhibition of ornithine aminotransferase (OAT) by 5-fluoromethylornithine rescues zebrafish from lethal concentrations of ammonium acetate and corrects hyperammonemia-induced biochemical alterations. Thus, OAT inhibition is a promising and effective therapeutic approach for preventing neurotoxicity and mortality in acute hyperammonemia.

## Introduction

Nitrogen is an essential building block of amino and nucleic acids in all living organisms. Protein ingested by food or derived from the body is the major source of excess nitrogen once nitrogen-containing compounds are used to build energy substrates. Deamination of amino acids liberates ammonium (NH_4_^+^). If present in increased concentrations, NH_4_^+^ is highly toxic to living organisms. Species have developed different strategies to dispose excess NH_4_^+^. While fishes (ammoniotelic organisms) excrete up to 90% of their nitrogenous waste directly into their aqueous environment, reptiles and birds save water by excreting uric acid (uricotelic organisms) [1]. Humans and terrestrial animals (ureotelic organisms) are unable to excrete NH_4_^+^ directly or to package large amounts of NH_4_^+^ into uric acid and metabolize NH_4_^+^ to water-soluble urea, an energy-dependent mechanism requiring three moles of ATP for each mole of urea.

The urea cycle contains five enzymes and two transporters that are involved in the irreversible detoxification of NH_4_^+^ to urea including carbamoylphosphate synthetase 1 (CPS1), ornithine transcarbamylase (OTC), argininosuccinate synthetase 1 (ASS1), argininosuccinate lyase (ASL), arginase 1 (ARG1), citrin or aspartate/glutamate carrier and the mitochondrial ornithine transporter 1 [2]. Furthermore, carbonic anhydrase VA and N-acetylglutamate synthase (NAGS) are required to form bicarbonate and N-acetylglutamate for the first enzymatic step, the formation of carbamoylphosphate.

Urea cycle disorders (UCDs) are caused by inherited deficiencies of the NH_4_^+^-detoxifying ureagenetic machinery. Estimated cumulative incidence of UCDs is between 1:35,000 to 1:50,000 newborns [3, 4]. UCD patients, except for those with citrin and ARG1 deficiency, characteristically present with hyperammonemic encephalopathy (HE), which often manifests as early as the first days of life, but can present with first symptoms at any age afterwards. Onset type and the peak plasma ammonium concentration of the initial manifestation correlate with clinical severity and outcome. The most severe manifestation is neonatal HE with coma; it is associated with a high risk of mortality and, in survivors, of severe neurologic dysfunction and intellectual disability [3, 5–11].

Maintenance treatment of UCDs is based on a low protein diet, supplementation with essential amino acids, citrulline and/or arginine, and application of nitrogen scavenging drugs (sodium benzoate, sodium and glycerol phenylbutyrate) as well as carglumic acid (for NAGS deficiency, partially responsive CPS1 deficiency and carbonic anhydrase VA deficiency). While low protein diet aims at reducing the production of nitrogenous waste, supplementation with citrulline (CPS1 and OTC deficiency, HHH syndrome) and/or arginine (for all UCDs except for NAGS and ARG1 deficiency) has been shown to stimulate the residual activity of the urea cycle in individuals with UCDs, maximizing ureagenesis and thus excretion of ammonium [2, 8]. Nitrogen scavengers bypass the urea cycle and thus exploit alternative pathways for excretion of waste nitrogen, thereby efficiently lowering plasma ammonium concentrations: Benzoate conjugates with glycine to generate hippurate and phenylacetate (following hepatic activation of its precursor phenylbutyrate) binds glutamine to form phenylacetylglutamine. Both conjugates, hippurate and phenylacetylglutamine, are water-soluble and renally excreted [2, 8]. For individuals with NAGS deficiency, partially responsive CPS1 deficiency and carbonic anhydrase VA deficiency administration of carglumic acid has been proven beneficial, as it mimics the function of N-acetylglutamate, the physiological activator of CPS1, stimulating the formation of carbamoylphosphate [2, 12]. During HE, treatment is intensified stepwise by implementation of intravenous nitrogen scavenging therapy and extracorporeal detoxification using hemodialysis [2, 8, 13]. Liver transplantation is a curative therapy for UCDs. Best outcome is achieved if liver transplantation is carried out between age 3–12 months and before irreversible brain damage manifests [14, 15]. One of the major concerns is timely identification and undelayed start of severity-adjusted therapy. For this purpose, some UCDs–mostly ASS1 and ASL deficiencies–have been included in newborn screening (NBS) programs in a few countries [3, 16]. Its effectiveness remains to be demonstrated, since hyperammonemic crises often manifest before NBS results are available [16].

Despite several diagnostic and therapeutic improvements over the last three decades, the outcome of UCD patients with neonatal onset is still poor and has only slightly improved–if at all [7]. Neonatal mortality in severe forms is often as high as one third to one half of affected individuals [2, 3, 6]. Therefore, a better understanding of the underlying pathophysiology and the identification of novel targets for therapy are urgently required to improve the clinical outcome of UCD patients.

Here we show that zebrafish, an organism physiologically lacking the urea cycle at later stages of embryonic development, is a suitable model to study the mechanisms of NH_4_^+^-induced toxicity and mortality and to investigate the efficacy of therapeutic interventions. Our finding that pharmacologic inhibition of ornithine aminotransferase (OAT) prevents mortality in zebrafish exposed to lethal NH_4_^+^ concentrations, opens new therapeutic avenues for patients with UCDs.

## Materials and methods

### Study approval

All experiments, particularly with regard to expected mortality rates in the survival and therapeutic studies, complied with local and international regulations and ethics guidelines and were approved by the local research ethics committee (Germany, Regierungspräsidium Karlsruhe, permit AZ 35–9185.81/G-85/16). The study was performed in compliance with ARRIVE guidelines [17].

### Zebrafish maintenance

Zebrafish were kept at 26 to 27°C in a 14 hours (h) light and 10 h dark cycle. Embryos were collected by natural spawning and raised at 28.5°C in E3 medium. The AB/AB wildtype strain was used for all experiments. Staging of embryos was done according to Kimmel et al. [18]. To inhibit pigmentation for subsequent in situ hybridization experiments, 0.2 mM 1-phenyl-2-thiourea was added at 1 day post fertilization (dpf).

### Model for acute hyperammonemia

To study acute hyperammonemia, zebrafish larvae (N = 100 per group and experiment) at different developmental stages (0 dpf to 4 dpf) were exposed to 5, 10 or 20 mM ammonium acetate (NH_4_Ac). As control for potential toxic effects of acetate, embryos were exposed to sodium acetate (NaAc) in parallel experiments using analogous concentrations. Survival was monitored by light microscopy every hour after start of exposure (NH_4_Ac or NaAc) for a maximum of 36 h using cessation of heartbeat as primary endpoint. To further delineate underlying disease mechanisms on the biochemical and molecular level, zebrafish embryos/larvae at developmental stages 1 dpf or 4 dpf were exposed to either 10 mM NH_4_Ac or NaAc for 6 or 12 h respectively, homogenized and subjected to subsequent analyses (qRT-PCR, amino acid analysis, quantification of 2-oxoglutarate, lactate or pyruvate). Since acute hyperammonemia in human neonates is associated with a high mortality rate of one third to one half of afflicted individuals and severe neurological sequelae in survivors [2, 3, 6], and to enable the delineation of underlying disease mechanisms including the efficacy testing of different therapeutic strategies with regard to survival, humane endpoints during the study period of 36 h were not defined. Mortality/survival rates are indicated in the respective results subsection using the Kaplan-Meier method. All exposed zebrafish embryos surving the observation period (36 h after start of exposure) were euthanized by addition of tricaine methanesulfonate (MS222) to the E3 medium to a final concentration of 300 mg/L. All scientific staff involved in zebrafish embryo handling and experiments have attended and successfully completed the “International Zebrafish and Medaka Course” at the Karlsruhe Institute of Technology, Germany, which is in compliance with the Federation for Laboratory Animal Science Associations (FELASA)—regulations and an equivalent of FELASA-B certificate.

### qRT-PCR

Embryos were anesthetized with 0.01% tricaine methanesulfonate, and RNA was obtained by standard TRIzol Reagent (Invitrogen)-based procedure. Equal amounts of RNA from each sample (500 to 800 ng) were used for cDNA synthesis using Maxima first strand cDNA synthesis kit (Thermo Fisher Scientific). PCR was performed on a CFX Connect^TM^ Real-Time cycler (Biorad) (denaturation step: 95°C for 25 sec, annealing and elongation step: 60°C for 30 sec) using SensiFast SYBR^TM^ Hi-ROX mix (Bioline) following the manufacturer’s instructions. The genes of interest were amplified with the following primers:

GLULA

GLULA-forward: 5’-TACTGACGGACACCCCTTTG-3’

GLULA-reverse: 3’-CAACCTGGAACTCCCACTGAG-5’

GLULB

GLULB-forward: 5’-CGAGGAAGGAGGTTTGAAGCATA-3’

GLULB-reverse: 3’-AGCCCTTCTTTTCCTGACCC-5’

GLULC

GLULC-forward: 5’-TCACAGAGTGCATTGCTTAGTG-3’

GLULC-reverse: 3’-CAGACCCTCTCCAGACCCAT-5’

GLSA

GLSA-forward: 5’-ATGACAAGAGAAGGAAGGCAG-3’

GLSA-reverse: 3’-GCACCGTCTGAAGTGGTTTTC-5’

GLS2B

GLS2B-forward: 5’-CGACTACTCGGGACAGTTCG-3’

GLS2B-reverse: 3’-CCAGTTCCTGACAGAAGCGA-5’

GPT

GPT-forward: 5’-GGCATAGCGTCAGTGTCCTT-3’

GPT-reverse: 3’-AACTGCTCCTGTAGGGTTGC-5’

GPT2

GPT2-forward: 5’-CTTGGAGGAGGGTGGAACAAA-3’

GPT2-reverse: 3’-ATCCTCTGGGAAACTAGGGCT-5’

OAT

OAT-forward: 5’-CGACCCGCATCAGTGTGAAC-3’

OAT-reverse: 3’-CGGACGCTCCTGTGTTTTAG-5’

GRIN1a

GRIN1a-forward: 5’-CATCCCAGGACGCCCAAT-3’

GRIN1a-reverse: 3’-CTCTTTCCTGCGTCCCGAAT-5’

GRIN1b

GRIN1b-forward: 5’-CCTCGACCAACTGTCCTTTGA-3’

GRIN1b-reverse: 3’-CGGCTTCGTCTTCACTTGC-5’

GRIN2ab

GRIN2ab-forward: 5’-CGAAGCAATGGAACGGTGTC-3’

GRIN2ab-reverse: 3’-AGGTCCATGAGGGTCTTTGC-5’

GRIN2da

GRIN2da-forward: 5’-GTAGGTTGGTGGGAGAACGG-3’

GRIN2da-reverse: 3’-AACCATGCTGTTGTTGAGCG-5’

GRIN3a

GRIN3a-forward: 5’-GGCTCCACACCAGTCAAAGATT-3’

GRIN3a-reverse: 3’-CCAGGTCATTTTGCCCCTCT-5’

The expression level of elongation factor 1-α was used for normalization.

### In situ hybridization (ISH) procedure

Specific antisense RNA probes were generated using a digoxigenin RNA labeling kit (Roche). Whole mount ISH of zebrafish larvae were carried out with standard procedures [19]. Corresponding sense probes were used as negative controls to confirm specificity of observed staining patterns. Stained embryos were mounted in glycerol for 24 h and imaged using a binocular microscope (MZ16 F, Leica).

### Preparation of whole embryo/larvae homogenates

Zebrafish embryos or larvae were washed three times with ice-cold phosphate-buffered saline (PBS) and homogenized with a pestle in an appropriate size to allow disruption in a 1.5 mL Eppendorf tube and additional sonification. Lysates were centrifuged at 13,000 × *g* at 4°C for 10 minutes (min). The supernatant was either subjected to downstream applications (e.g. amino acid analysis, quantitative analysis of 2-oxoglutarate or pyruvate and lactate) or stored at -80°C until use. Protein concentrations were determined according to a modified Lowry protocol [20, 21] using bovine serum albumin as a standard.

### Amino acid analysis

Amino acid content in whole embryo or larvae homogenates was quantitatively analyzed by high-performance liquid chromatography (HPLC). The amino acid concentrations of each sample were normalized to its protein content.

### Quantitative analysis of 2-oxoglutarate

2-Oxoglutarate was determined in whole embryo or larvae homogenates using a commercially available colorimetric 2-oxoglutarate assay kit (Abcam) according to the manufacturer’s instructions. In this assay, 2-oxoglutarate is transaminated to pyruvate, which then is utilized to convert the optical density of a nearly colorless probe, thereby generating a colorimetric signal proportional to the 2-oxoglutarate content. Briefly, following washing with ice-cold PBS, control or NH_4_Ac-exposed larvae were homogenized in 2-oxoglutarate assay buffer and centrifuged for 10 min at 13,000 × *g* at 4°C in an Eppendorf centrifuge to remove insoluble material. The supernatant was transferred to a new Eppendorf tube and subjected to deproteinization using perchloric acid (PCA) in a final concentration of 1 M. To precipitate excess PCA and adjust the pH in the range of 6.5 to 8.0, ice-cold potassium hydroxide (2 M) was added. After centrifugation for 15 min at 13,000 × *g* at 4°C, the supernatant was added to a 2-oxoglutarate converting enzyme mix and incubated for 30 min at 37°C in the dark followed by colorimetric readout (optic density at a wavelength of 570 nm) using a SpectraMax Plus 384 microplate reader (Molecular Devices). To control for background signal, samples were additionally subjected to colorimetric readout in the absence of 2-oxoglutarate converting enzyme. Sample background values were subtracted from each sample reading for correction. The amount of 2-oxoglutarate was determined by extrapolation from a respective standard curve. For data consistency, measurements were performed in duplicates for each experimental series. 2-Oxoglutarate concentrations were normalized to the protein content.

### Quantitative analysis of pyruvate and lactate

Pyruvate and lactate concentrations in homogenates of control or NH_4_Ac-exposed larvae at 4 dpf were determined using a colorimetric enzymatic assay. Briefly, pyruvate content was analyzed by addition of NADH and lactate dehydrogenase to the homogenate using the lactate dehydrogenase reaction, which results in the formation of lactate and NAD^+^. Pyruvate concentrations were quantitatively determined by the reduction of NADH concentrations at 340 nm using an Olympus AU400 chemistry analyzer. Accordingly, lactate content was quantified using the lactate dehydrogenase reaction in reverse by measuring the increase of NADH concentrations at the same wavelength. Pyruvate and lactate concentrations were normalized to the protein content in each sample.

### Treatments

Zebrafish larvae at 4 dpf were preincubated for 30 min with 25 μM L-methionine sulfoximine (L-MSO; Sigma Aldrich), 50 μM ketamine (Sigma Aldrich) or 50 μM memantine (Sigma Aldrich) alone or in combination, or 5-fluoromethylornithine (5-FMO; ChemSpace) in a dose range from 50 to 200 μM. Hereafter, larvae were exposed to 10 mM NH_4_Ac for 36 h and their survival monitored. For amino acid analyses or analysis of mRNA expression levels of NMDA receptor subunits, zebrafish larvae treated with or without 5-FMO in a dose range from 50 to 200 μM were homogenized 12 h after start of NH_4_Ac exposure and subjected to HPLC or RNA extraction followed by qRT-PCR analysis. For quantification of phenotypic presentation, zebrafish larvae at developmental stage 4 dpf treated with or without 200 μM 5-FMO were exposed to 10 mM NH_4_Ac for 12 h. Larvae were fixed in 5% PFA in PBS at 4°C overnight and imaged using a binocular microscope (MZ16 F, Leica). The exposed cohorts with or without preincubation with 200 μM 5-FMO were compared to a non-exposed control group.

### Statistical analysis

Data are expressed as mean +/- SD unless otherwise stated. Experiments were performed at least in triplicates. Differences between mean values of groups were evaluated by two-tailed Student’s t-test (two groups of samples) or ANOVA with a Tukey-Kramer post-test (multiple groups of samples) using the Prism software (GraphPad Software, La Jolla, CA, USA). Differences in survival rates were analyzed applying the log-rank test. P values <0.05 were considered significant.

## Results

### Ammonium acetate-induced mortality is age- and concentration-dependent

**Fig 1:**
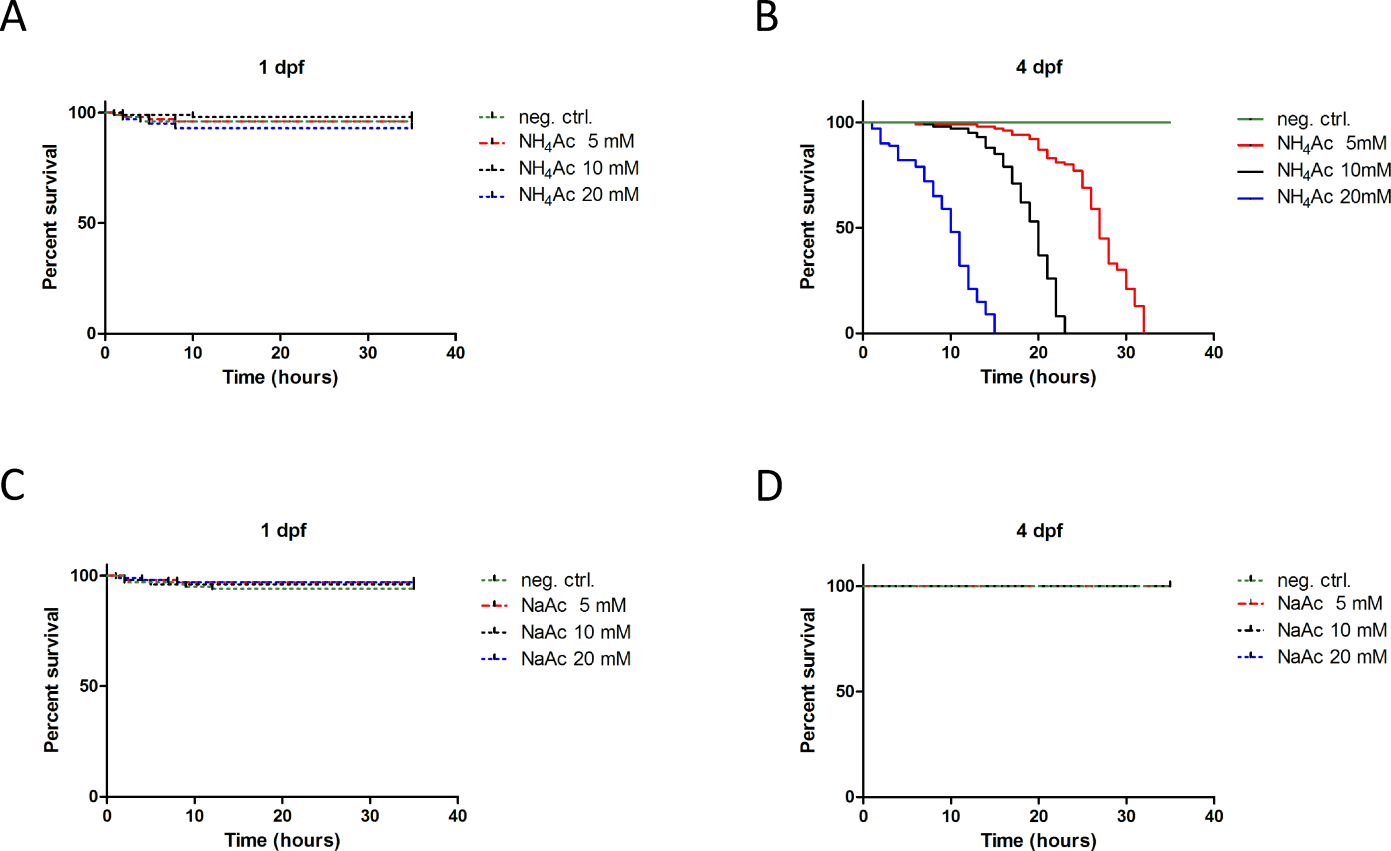
Kaplan-Meier plots of ammonium acetate-induced mortality. Zebrafish larvae (n = 100 per group) at 1 or 4 dpf were exposed to varying concentrations (5, 10 and 20 mM) of either NH_4_Ac or NaAc and survival rates monitored for up to 36 h. While NH_4_Ac was not toxic to zebrafishes at 1 dpf (A), exposure at 4 dpf induced death of all larvae in a dose-dependent manner (log-rank test, P<0.001) (B). In contrast, NaAc had virtually no toxic or lethal effect at either developmental stage (C, D).

To assess whether developing zebrafish embryos are sensitive to NH_4_^+^ we exposed them at different stages of embryonic development to varying concentrations of NH_4_Ac and monitored survival during 36 h after start of exposure. Treatments of zebrafish larvae up to 1 dpf did not affect embryonic development or survival rates at any NH_4_Ac concentration (5 to 20 mM) tested (**Fig 1A**). Similarly, embryos treated at 2 or 3 dpf were not affected (**S1 Fig**). In contrast, exposure at 4 dpf to NH_4_Ac, induced death of all larvae (**Fig 1B**). Hyperammonemia-induced mortality was concentration-dependent (log-rank test, P<0.001). NH_4_Ac-exposed larvae died within 32 h (5 mM NH_4_Ac), 23 h (10 mM NH_4_Ac) and 15 h (20 mM NH_4_Ac), respectively, and showed a median survival of 27 h (5 mM NH_4_Ac), 20 h (10 mM NH_4_Ac) and 15 h (20 mM NH_4_Ac), respectively (**Fig 1B**). In contrast, NaAc was not toxic to zebrafish larvae at any developmental stage (**Fig 1C and 1D**) excluding that the acetate compound contributes to the observed toxicity.

### Acute hyperammonemia induces expression of glutamine synthetase and glutaminase

As NH_4_Ac-mediated toxicity and mortality was evident not before 4 dpf, we hypothesized that developmental alterations establishing a genetic or metabolic cascade, which is sensitive to NH_4_^+^, are responsible for the observed differences. Since cerebral glutamine-glutamate cycle is known to play a pivotal pathophysiological role in hyperammonemic conditions in humans [22] and was shown to be activated in a rat model of acute liver failure [23], we investigated whether glutamine synthetase and glutaminase were involved in NH_4_Ac-induced mortality. In zebrafish, three paralogs of glutamine synthetases, i.e. GLULA, GLULB and GLULC, have been described [24], while four different isoforms of glutaminases, i.e. GLSA, GLSB, GLS2A and GLS2B, are known. Since the glutaminase isoform *Glsa* is selectively expressed in brain, whereas *Gls2b* is specifically expressed in the liver and the intestinal bulb, with expression starting at age 3 dpf in in both [25], we have focused on these two isoforms.

**Fig 2:**
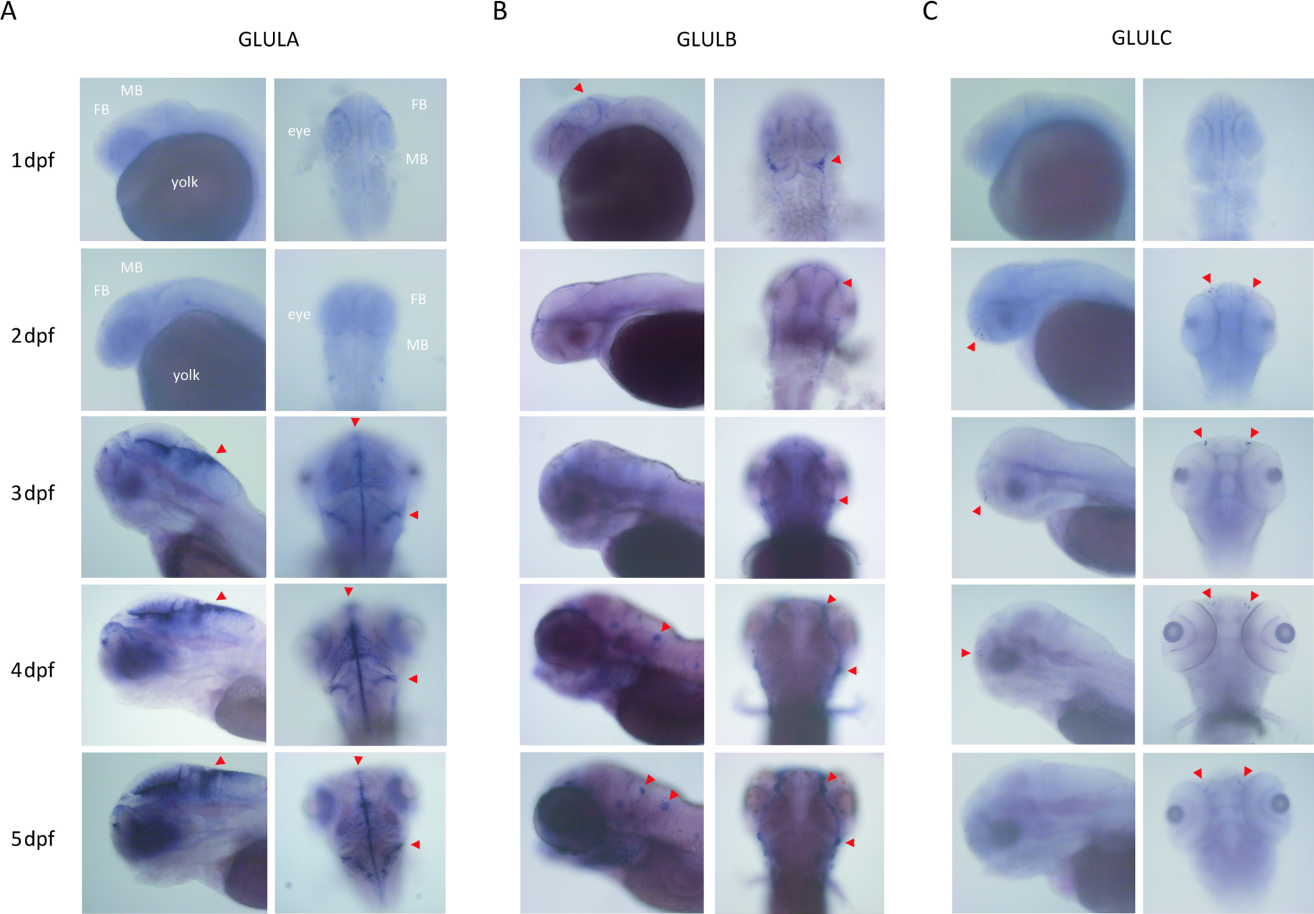
Cerebral expression patterns of glutamine synthetase isoforms during zebrafish development. Localization of mRNAs encoding for the glutamine synthetases GLULA, GLULB and GLULC in the CNS of zebrafish larvae as visualized by ISH. Pictures are representative images of 3 independent experiments (n = 50 embryos/larvae per stage and experiment). Images show the embryonic heads at stages indicated. Left columns of each row show latter with anterior to the left and right columns with anterior to the top; FB, forebrain; MB, midbrain. *Glula* was expressed in radial glia (arrowheads) starting at 3 dpf with constantly increasing expression up to 5 dpf (A). *Glulb* was expressed in neural crest and neuromast cells (arrowheads) (B), *Glulc* expression was restricted to distinct cells of the nostrils (arrowheads) throughout indicated stages (C).

To further address the relevance of glutamine synthetase isoforms, we performed a temporal and spatial expression analysis in the central nervous system (CNS) during zebrafish development using whole mount ISH. Expression of glutamine synthetase isoform *Glula* in radial glial cells [24] appeared not before 3 dpf with a further increase until 5 dpf, and thus parallels the observed hyperammonemia-induced neurotoxicity and mortality (**Fig 2A**). The paralog *Glulb* is expressed in distinct cells of the CNS at 1 dpf but remains to be restricted to peripheral parts of the midbrain after 3 dpf. Moreover, strong expression can be detected in neuromast cells and to a lower extent in defined parts of the CNS up to 5 dpf [24] (**Fig 2B**). In contrast, *Glulc* is absent from the CNS and only expressed in distinct cell types of the nostrils (**Fig 2C**). Representative images of negative control stainings using the respective *Glula*, *Glulb* and *Glulc* sense-probes are illustrated in **S2 Fig**. Results of the temporal (overall) expression analysis of glutamine synthetases *Glula*, *Glulb* and *Glulc* as well as glutaminase isoforms *Glsa* and *Gls2b* are depicted in **S3 Fig**.

**Fig 3:**
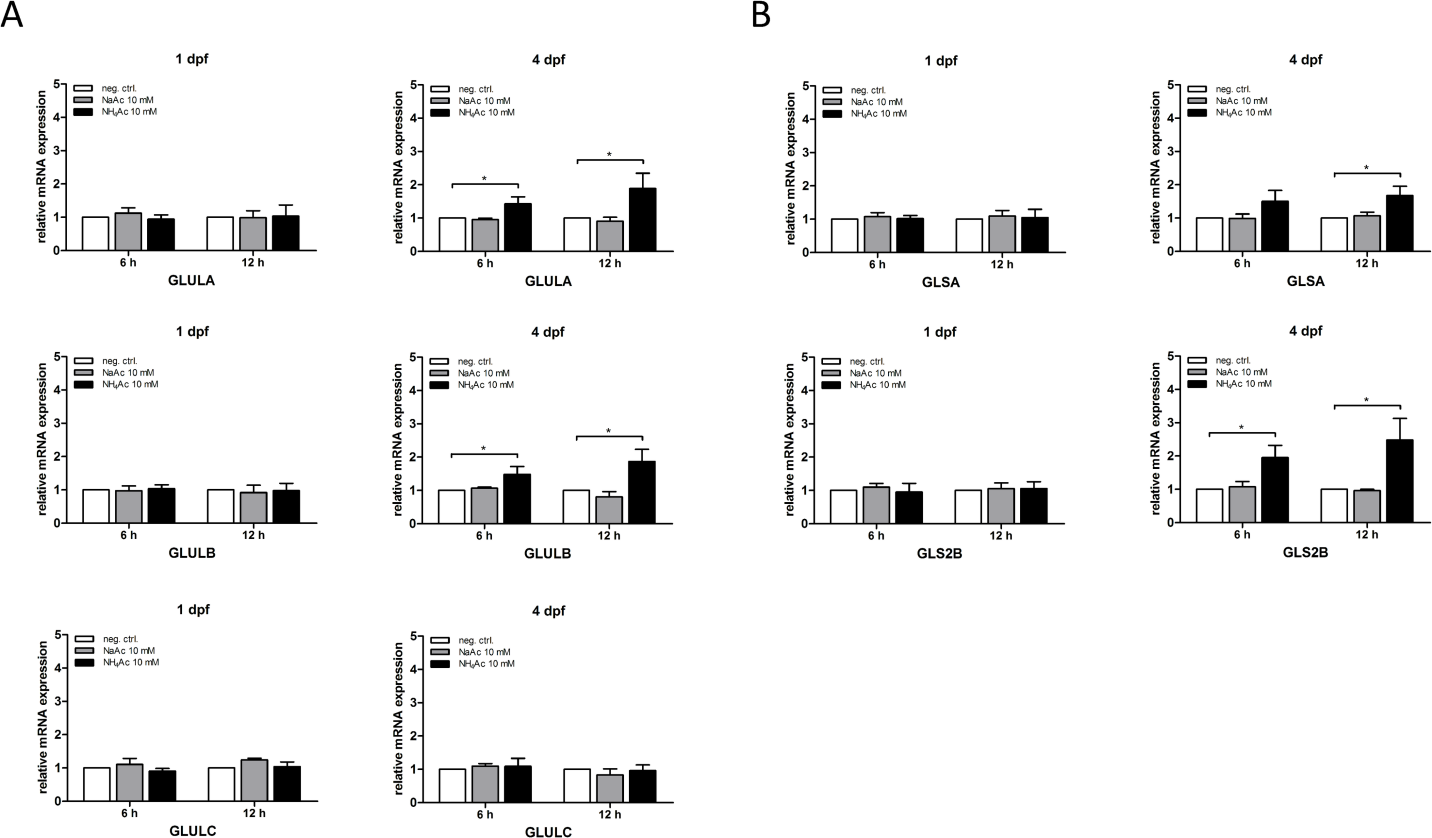
Acute hyperammonemia induces expression of specific glutamine synthetase and glutaminase isoforms. Zebrafish larvae at 1 or 4 dpf were exposed to 10 mM of either NH_4_Ac or NaAc and expression of indicated mRNAs analyzed using qRT-PCR. While NH_4_^+^-exposure did not change expression of glutamine synthetase (*Glula*, *Glulb* and *Glulc*) or glutaminase (*Glsa*, *Gls2b*) isoforms at 1 dpf, exposure at 4 dpf induced gene expression of *Glula*, *Glulb*, *Glsa* and *Gls2b*, respectively (A, B). Data are expressed as mean +/- SD in fold-change (whole body lysates, n = 3 with 50 larvae per group and experiment; ANOVA, *P<0.05).

Interestingly, while there was no induction of gene expression of glutamine synthases or glutaminases evident in exposed zebrafish larvae at 1 dpf as judged by qRT-PCR experiments, expression of *Glula*, *Glulb* as well as *Glsa* and *Gls2b* was significantly increased in NH_4_^+^-exposed larvae at 4 dpf compared to untreated and NaAc-exposed controls (**Fig 3A and 3B**).

### Acute hyperammonemia leads to massive increase in glutamate and glutamine concentrations and depletion of alanine and ornithine

**Fig 4:**
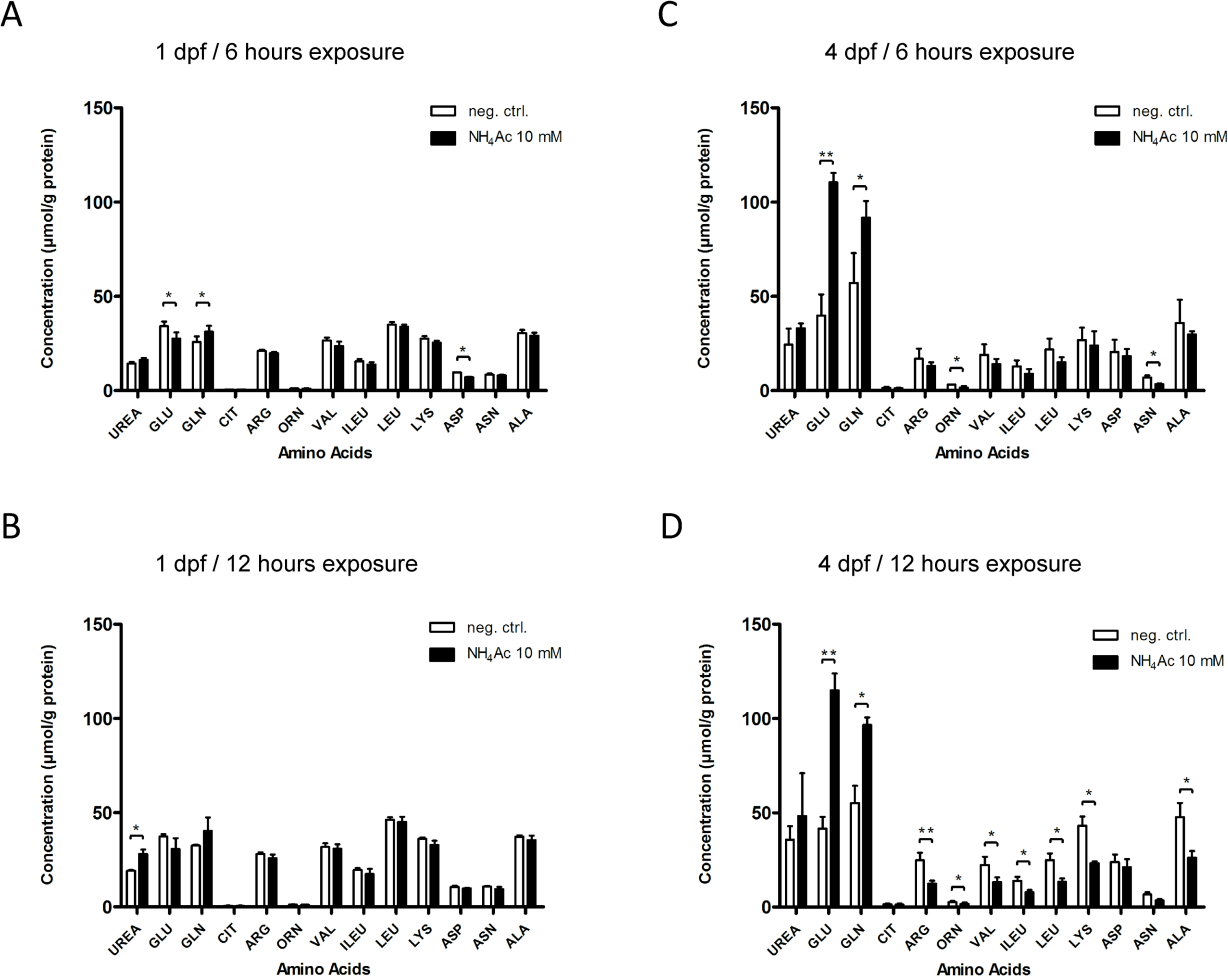
Exposure to NH_4_Ac leads to strong increase in glutamine and glutamate concentrations and depletion of ornithine and alanine. Zebrafish larvae at 1 or 4 dpf were exposed to 10 mM NH_4_Ac for 6 or 12 h and amino acids content in whole body lysates determined by HPLC. While larvae at 1 dpf showed increased urea production after 12 h of NH_4_-exposure when compared to the non-exposed control cohort (B), exposed zebrafishes at 4 dpf exhibited significantly increased glutamine and glutamate concentrations after 6 and 12 h, respectively (C, D). Simultaneously, NH_4_-exposure caused a significant depletion of branched-chain amino acids, alanine, ornithine and subsequently arginine at 4 dpf (D). Data are expressed as mean +/- SD in μmol per g protein (whole body lysates, n = 3 with 50 larvae per group and experiment; Student’s t-test, *P<0.05, **P<0.01).

Next, we studied the effects of acute hyperammonemia on amino acids by HPLC. In larvae at 1 dpf, NH_4_Ac exposure (10 mM) for 6 h caused a decrease in glutamate with concomitant increase in glutamine concentrations (glutamate/glutamine ratio: 0.88) demonstrating increased glutamine synthesis from glutamate as initial adaptation to acute hyperammonemia. Other amino acid levels remained unaltered. Notably, glutamine and glutamate concentrations gradually normalized after 12 h of NH_4_Ac exposure, while urea formation was increased when compared to the control cohort at this developmental age (**Fig 4A and 4B**).

In striking contrast to the biochemical changes observed in zebrafish larvae at 1 dpf, exposed larvae at 4 dpf exhibited simultaneously elevated glutamate and glutamine concentrations (glutamate/glutamine ratio: 1.20) after 6 and 12 h exposure, respectively. In addition, hyperammonemia caused a depletion of essential branched-chain amino acids, as well as alanine, ornithine and subsequently arginine levels (**Fig 4C and 4D**).

### Transamination via ornithine and alanine aminotransferases are pivotal processes in acute hyperammonemia

Since glutamine synthesis is the only detoxification strategy of the brain for the detoxification of ammonium [26, 27] which is characterized by the condensation of glutamate and ammonium to form glutamine, massively elevated glutamate concentrations as observed in our experiments were unexpected. As increased glutamate concentrations in acute hyperammonemia were associated with depletion of alanine and ornithine, we hypothesized that transamination processes via alanine and ornithine aminotransferases were responsible for the observed biochemical changes. In zebrafish, two homologs of alanine aminotransferases, GPT and GPT2, have been annotated [28, 29], while for ornithine aminotransferase only one single isoform, OAT, has been predicted by genome sequence analysis (NM_001317170.1). To further address our hypothesis, we performed a temporal expression analysis of alanine and ornithine aminotransferases during zebrafish development. Interestingly, while *Gpt* was expressed with only mild variation throughout whole embryonic development, *Gpt2* exhibits strong maternal expression with a decrease at 75% epiboly. In contrast, *Oat* expression constantly increased during neurulation, peaking at 5 dpf (**S4 Fig**).

**Fig 5:**
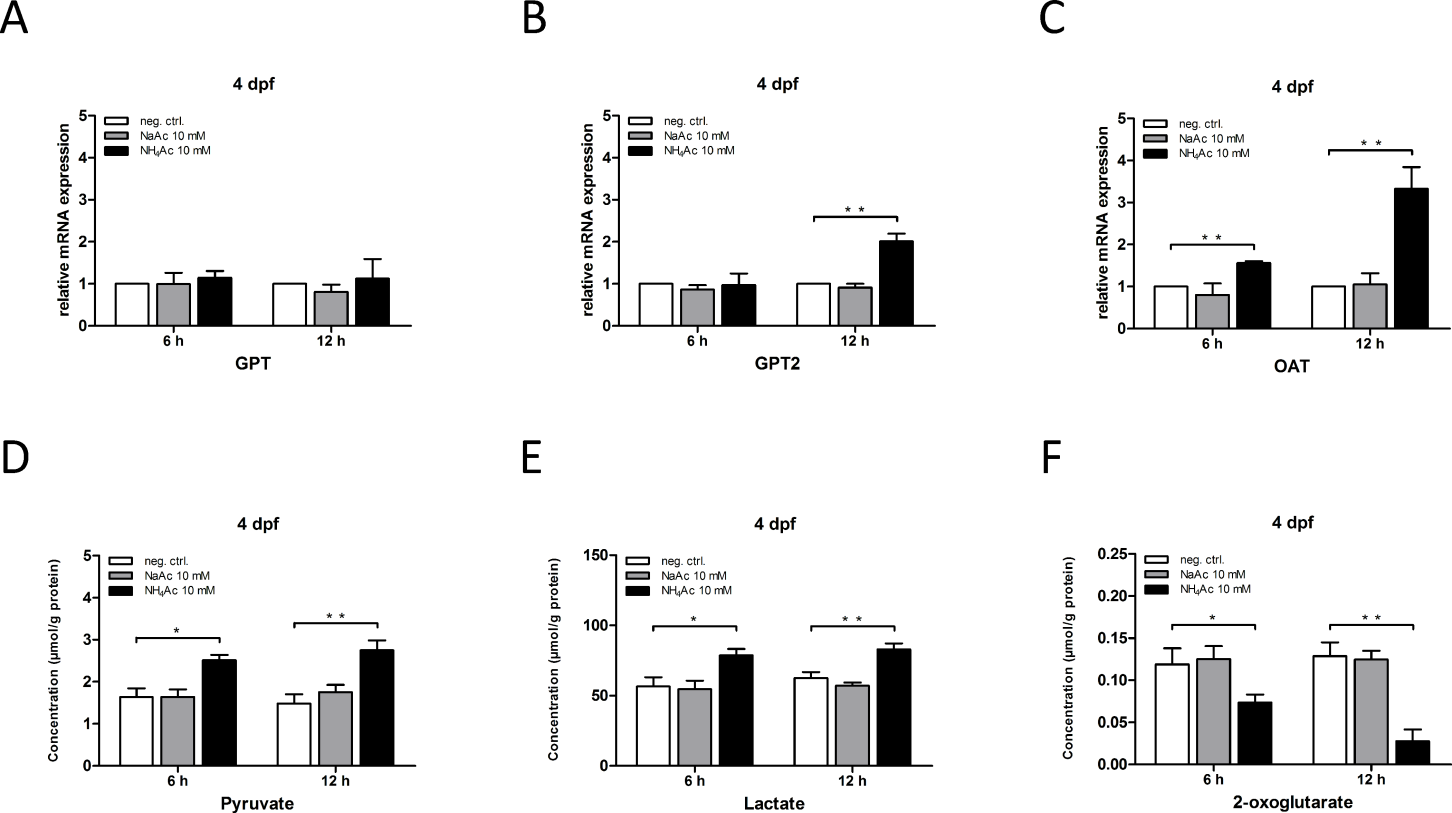
Transamination via ornithine and alanine aminotransferases are pivotal processes in acute hyperammonemia. Zebrafish larvae at 4 dpf were exposed to 10 mM NH_4_Ac or NaAc for 6 or 12 h and expression of indicated mRNAs analyzed using qRT-PCR. Whole body lysates of exposed zebrafish embryos were subjected to quantitative analysis of pyruvate, lactate or 2-oxoglutarate concentrations as described under *Materials and Methods*. NH_4_-exposure induced gene expression of alanine aminotransferase isoform *Gpt2* and *Oat*, while *Gpt* expression was unchanged (A-C). Exposure to NaAc had no effect on the expression of either aminotransferase (A-C). Larvae exposed to NH_4_Ac displayed increased pyruvate and lactate concentrations when compared to the control cohort (D, E). In addition, 2-oxoglutarate concentrations were significantly depleted upon NH_4_-exposure after 6 h, with a further decline after 12 h (F). Data are expressed as mean +/- SD in fold-change (A-C) or μmol per g protein (D-F) (whole body lysates, n = 3 with 50 larvae per group and experiment; ANOVA, *P<0.05, **P<0.01).

Subsequently, we analyzed the regulation of gene expression of aminotransferases under hyperammonemic conditions. While mRNA expression of *Gpt* was unaltered in zebrafish larvae exposed at 4 dpf, *Gpt2* expression was significantly increased after 12 h of NH_4_Ac exposure (10 mM) (**Fig 5A and 5B**). Interestingly, exposed zebrafish larvae already exhibited a significant increase in *Oat* expression after 6 h, with a further elevation up to 3.5-fold after 12 h as compared to non-exposed control (**Fig 5C**). These findings highlight an adaptive regulatory gene induction of the ammonium-metabolizing transaminases *Gpt2* and *Oat*. While GPT2 reversibly catalyzes the transfer of an amino group from alanine to 2-oxoglutarate to form glutamate and pyruvate, OAT generates glutamate and Δ1-pyrroline-5-carboxylate by transfer of the delta-amino group of ornithine to 2-oxoglutarate [30, 31].

To confirm that the activities of GPT2 and OAT are indeed functionally relevant for the observed changes in amino acid patterns (e.g. glutamate increase, depletion of alanine and ornithine), we investigated pyruvate, lactate and 2-oxoglutarate concentrations upon NH_4_Ac exposure. As expected, acute hyperammonemia caused a significant increase of both pyruvate and lactate. In addition, 2-oxoglutarate concentrations massively depleted in zebrafish larvae exposed to NH_4_Ac at 4 dpf (**Fig 5D, 5E and 5F**).

### Glutamatergic signaling via NMDA receptors is mediating neurotoxicity in acute hyperammonemia

**Fig 6:**
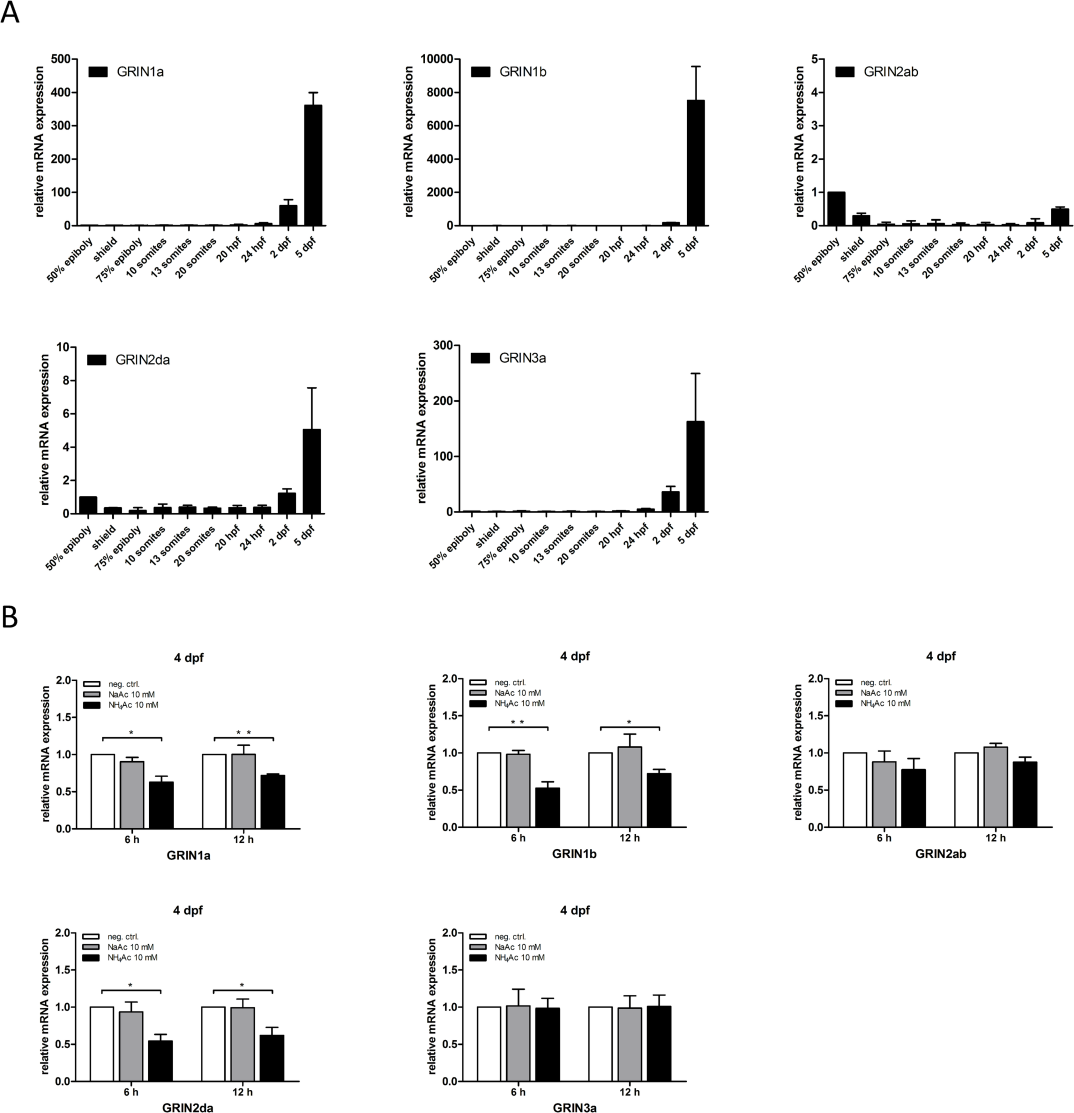
Glutamatergic signaling via NDMA receptors mediates neurotoxicity in acute hyperammonemia. Endogenous mRNA expression of zebrafish homologs of NDMA receptor subunits (*Grin1a*, *Grin1b*, *Grin2ab*, *Grin2da* and *Grin3a*) during zebrafish development (50% epiboly to 5 dpf). Expression of *Grin1a*, *Grin1b*, *Grin2da* and *Grin3a* strongly increased at 5 dpf (A), coinciding with the time point of NH_4_-induced mortality. Larvae exposed to 10 mM NH_4_Ac at 4 dpf displayed a downregulated gene expression of NMDA receptor subunits *Grin1a*, *Grin1b* and *Grin2da* after 6 and 12 h, respectively (B). Data are expressed as mean +/- SD in fold-change (whole body lysates, n = 3 with 50 larvae per group and experiment; ANOVA, *P<0.05, **P<0.01).

Since pathologically increased glutamate concentrations in the brain are known to induce neurotoxicity mainly via excessive activation of NMDA receptors [32], we further investigated the effect of acute hyperammonemia on the expression levels of specific NMDA receptor subunits. In zebrafish, five different NMDA receptor subunits, i.e. GRIN1a, GRIN1b, GRIN2ab, GRIN2da and GRIN3a, have been described [33]. In the first step, we analyzed the temporal expression of NMDA receptor isoforms during zebrafish development on the mRNA level. While *Grin1a*, *Grin1b*, *Grin2da* and *Grin3a* were hardly expressed at early developmental stages, their expression strongly increased at 5 dpf (**Fig 6A**) coinciding with the time point of hyperammonemia-induced neurotoxicity and mortality. In acute hyperammonemia, zebrafish larvae exposed to NH_4_Ac at 4 dpf displayed a significant downregulation of the gene expression of NMDA receptor subunits *Grin1a*, *Grin1b* and *Grin2da* (**Fig 6B**), most likely accounting for a compensatory mechanism to reduce the toxic effects of elevated glutamate concentrations in brain.

### OAT inhibition prevents neurotoxicity and mortality in acute hyperammonemia

**Fig 7:**
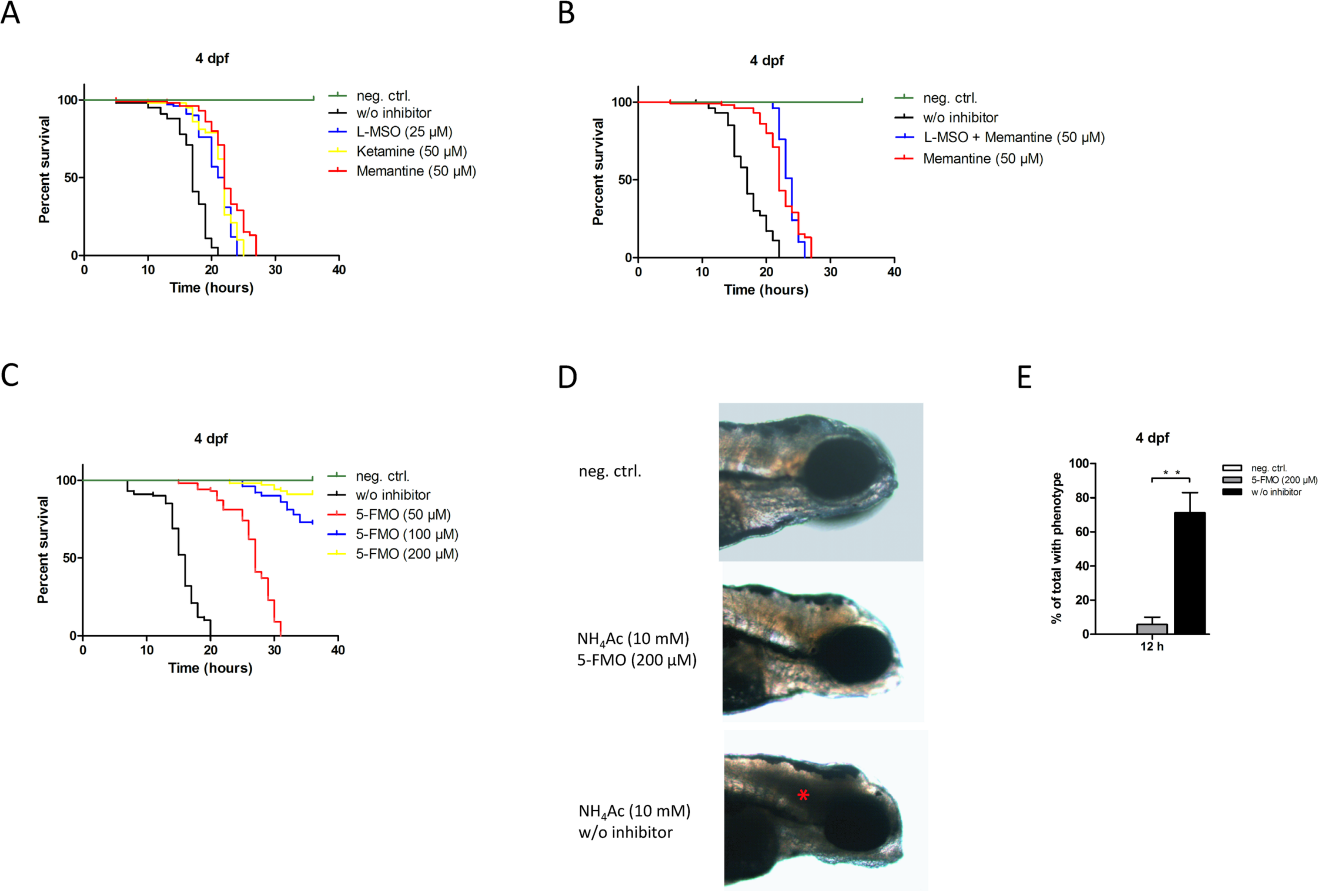
Comparison of different therapeutic concepts: OAT-inhibition prevents mortality in acute hyperammonemia and rescues brain cell death. Zebrafish larvae (n = 100 per group) at 4 dpf were treated with L-MSO (25 μM), ketamine (50 μM), or memantine (50 μM) alone or in combination or with increasing concentrations of 5-FMO (50 to 200 μM) for 30 minutes following exposure to 10 mM NH_4_Ac. While mock-treated zebrafish larvae died within 21 h (median survival: 17 h), larvae preincubated with L-MSO or ketamine succumbed to 10 mM NH_4_Ac within 24 h (median survival: 21.5 h; log-rank test, P<0.01) or 25 h (median survival: 22 h; log-rank test, P<0.01), respectively. Treatment with memantine prolonged survival up to 27 h (median survival 22h; log-rank test, P<0.01) (A). Combination of L-MSO and memantine did not further increase survival rates as compared to the treatment with memantine alone (B). Treatment with 50 μM 5-FMO for 30 min prior to NH_4_-exposure prolonged survival up to 31 h (median survival: 27 h; log-rank test, P<0.0001). Further dosage escalation had an incremental benefit on survival rates. While 72% of larvae treated with 100 μM 5-FMO survived the observation period of 36 h of NH_4_Ac exposure, treatment with 200 μM 5-FMO increased the survival rate of exposed larvae to 91% (C). Representative illustration of zebrafishes at 4 dpf either treated with 200 μM 5-FMO or mock (w/o inhibitor) for 30 min prior to exposure to 10 mM NH_4_Ac for 12 h (n = 3 with 50 larvae per group and experiment) (D). Zebrafishes were fixed with 5% PFA in PBS overnight and imaged using a binocular microscope (MZ16 F, Leica). Pictures show larvae with anterior to the right. Exposure to 10 mM NH_4_Ac induced brain cell death (asterix), which was rescued by treatment with 5-FMO in a concentration of 200 μM as compared to the non-exposed control cohort (D). Quantitative analysis of larvae exhibiting signs of brain damage in the different groups (E). Data are expressed as mean +/- SD in % of total exhibiting microscopic signs of brain cell death (n = 3 with 50 larvae per group and experiment; ANOVA, **P<0.01).

Since glutamine synthesis and NMDA receptor-mediated signaling is thought to play an important role in hyperammonemia-induced neurotoxicity and since our experiments unraveled hyperammonemia-induced changes in the expression pattern of these components, we wondered whether their therapeutic modulation could protect zebrafish larvae against NH_4_Ac-induced toxicity and death. Therefore, we next investigated the therapeutic efficacy of pharmacologic inhibition of glutamine synthesis or/and NMDA receptor signaling in acute hyperammonemia using the glutamine synthetase inhibitor L-MSO and the NMDA receptor antagonists memantine and ketamine, alone or in combination. However, treatment with either of the compounds had only limited beneficial effects on survival rates: While mock-treated zebrafish larvae at 4 dpf died within 21 h (median survival: 17 h), larvae preincubated with 25 μM L-MSO or ketamine succumbed to 10 mM NH_4_Ac within 24 h (median survival: 21.5 h) or 25 h (median survival: 22 h), respectively. Treatment with 25 μM memantine was more beneficial for survival rates (time to total lethality: 27 h; median survival: 22 h) when compared to L-MSO or ketamine (**Fig 7A**). The combination of L-MSO and memantine did not further increase survival rates as opposed to the treatment with memantine alone (**Fig 7B**).

To assess whether OAT inhibition might be a more effective therapeutic strategy against hyperammonemia-induced toxicity and mortality, we tested 5-FMO, a specific and irreversible OAT inhibitor [34]. Indeed, zebrafish larvae at 4 dpf precincubated with 50 μM 5-FMO 30 min prior to NH_4_Ac exposure survived significantly longer as opposed to the mock-treated cohort (**Fig 7C**). While mock-treated zebrafish larvae succumbed to 10 mM NH_4_Ac within 20 h (median survival: 16 h), 50 μM 5-FMO prolonged survival up to 31 h (median survival: 27 h; log-rank test, P<0.0001). In addition, further dosage escalation of 5-FMO had an incremental benefit on the survival rates of the treated zebrafish cohorts. While 72% of larvae preincubated with 100 μM 5-FMO survived the observation period of 36 h of NH_4_Ac exposure, the survival rate of larvae treated with 200 μM 5-FMO was further increased to 91% (**Fig 7C**). Furthermore, treatment with 200 μM 5-FMO rescued hyperammonemia-induced brain cell death as determined by light microscopy (**Fig 7D**). Whereas in the mock-treated cohort 71% of larvae exhibited morphological signs of brain damage 12 h after exposure to 10 mM NH_4_Ac, the phenotypic occurrence was reduced to 6% by treatment with 200 μM 5-FMO (**Fig 7E**).

**Fig 8:**
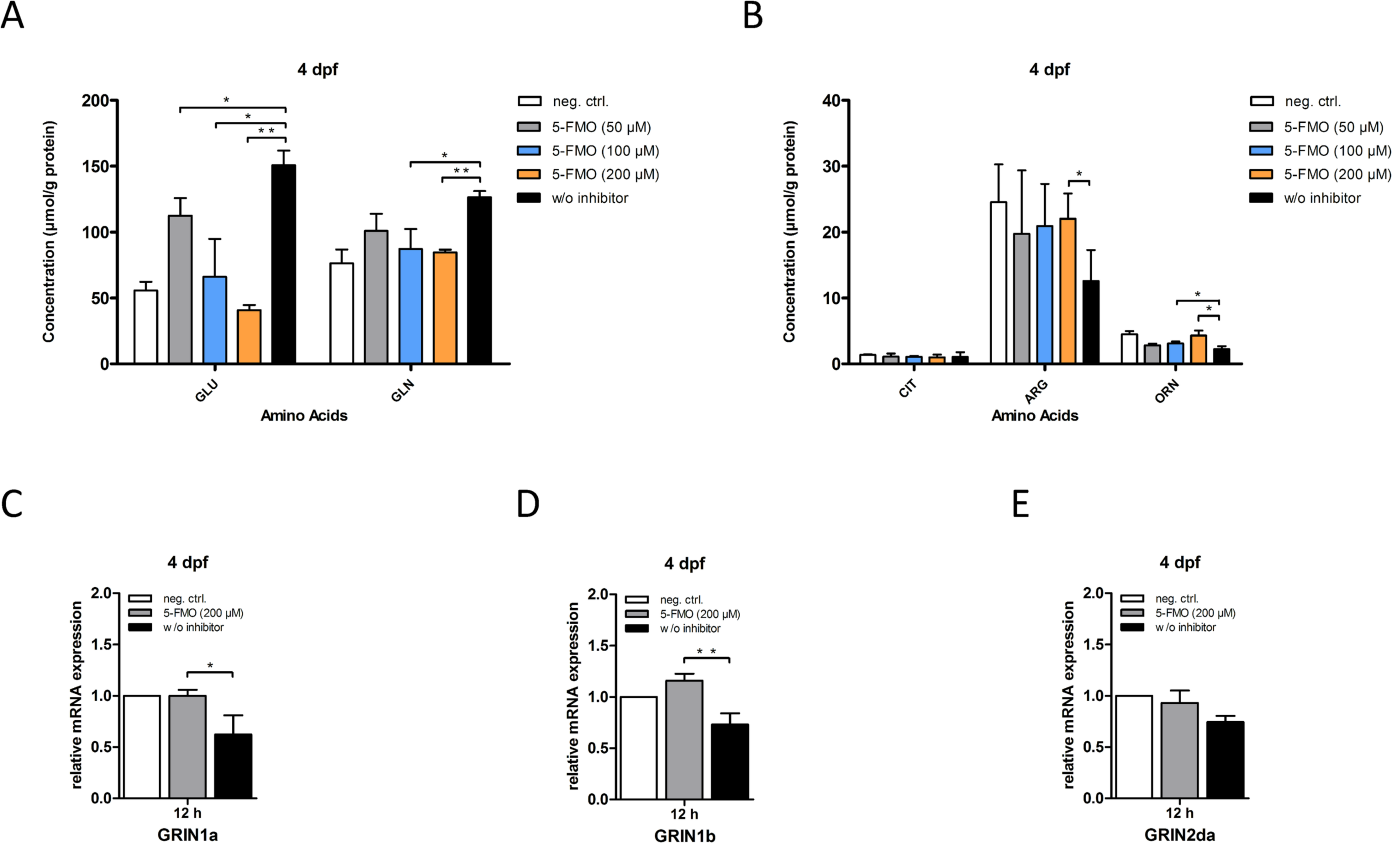
OAT-inhibition corrects biochemical alterations and NMDA receptor expression in acute hyperammonemia. Zebrafish larvae at 4 dpf were treated with increasing concentrations of 5-FMO (50 μM to 200 μm) for 30 min followed by exposure to 10 mM NH_4_Ac for 12 h. Thereafter, zebrafishes were lysed and either subjected to HPLC for determination of amino acids (A, B) or RNA extraction and subsequent qRT-PCR analysis (C-E). Treatment with 5-FMO led to a normalization of glutamate and glutamine concentrations (A) and restored ornithine and arginine concentrations (B) in a dose-dependent manner. Moreover, mRNA expression levels of NMDA receptor subunits *Grin1a* and *Grin1b* normalized upon treatment with 200 μm 5-FMO (C, D). Data are expressed as mean +/- SD in μmol per g protein (A, B) or in fold-change (C-E) (whole body lysates, n = 3 with 50 larvae per group and experiment; ANOVA, *P<0.05, **P<0.01).

Notably, in acute hyperammonemia treatment with 5-FMO restored ornithine and arginine concentrations and led to a normalization of glutamate and glutamine concentrations (**Fig 8A and 8B**) with subsequent correction of *Grin1a* and *Grin1b* receptor expression (**Fig 8C and 8D**). These results clearly indicate that OAT inhibition is an effective and promising therapeutic strategy preventing neurotoxicity and mortality in a zebrafish model of acute hyperammonemia.

## Discussion

In this study, we established a zebrafish model for acute hyperammonemia by exposing zebrafish larvae to defined concentrations of NH_4_Ac. Acute hyperammonemia led to a transamination-dependent increase of osmolytic glutamine and glutamate concentrations and decreased mRNA expression for different NMDA receptor subunits, which suggests that hyperammonemia induces neurotoxicity and death via excessive activation of NMDA receptors. Inhibition of OAT using 5-FMO corrected biochemical alterations and significantly prolonged survival rates of exposed zebrafish larvae, while none of the other therapeutic interventions, i.e. inhibition of glutamine synthetase (L-MSO) and NMDA receptors (ketamine, memantine), was able to rescue ammonium-exposed zebrafish larvae.

Zebrafish belongs to a subgroup of teleost fish species that are capable of producing significant levels of urea during early embryonic development owing to the presence of a functional urea cycle, but become ammoniotelic with further developmental age [35, 36]. In developing zebrafish larvae, the five enzyme transcripts necessary for ureagenesis (CPS1, OTC, ASS1, ASL and ARG1) are co-expressed in the embryonic endoderm adjacent to the anterior part of the yolk sac and the floor plate from 5 hpf up to 48 hpf [36]. Ureagenesis and urea excretion peaks at 48 hpf and is reduced significantly thereafter, while direct ammonium excretion increases constantly after hatching at 2 to 3 dpf [35, 36]. Injections of a morpholino targeting the rate-limiting enzyme CPS1 led to a significant reduction of urea excretion and whole urea body content in the injected zebrafish larvae up to 48 hpf, demonstrating that ureagenesis indeed is performed by a functional urea cycle in early embryonic development [36]. Consistently, we found that exposure of zebrafish larvae with 10 mM NH_4_Ac at 24 hpf but not at subsequent stages significantly triggered ureagenesis. This result supports the idea of a temporarily active urea cycle before hatching. After hatching zebrafish larvae become primarily ammoniotelic with direct excretion of NH_4_^+^ ions into the aqueous environment. This adaptation is achieved by the timely and spatial expression of proteins belonging to the family of membrane-bound rhesus (Rh) glycoproteins. The proteins, first described in erythrocytes, increase the ammonium permeability of the plasma membrane and act as specific ammonium transporters [37]. The members of the Rh protein family known to transport ammonium across the cell membrane are Rh-associated glycoprotein (Rhag), Rh B glycoprotein (Rhbg) and Rh C glycoprotein (Rhcg). The expression of those three ammonium transporters constantly increases during zebrafish embryonic development peaking between 3 and 4 dpf [34]. All three transporters localize to the gill filaments and distinct cells on the outer surface of the yolk sac at 4 dpf, the latter suggesting the presence of Rh glycoproteins within a specific cell type on the skin [35]. Morpholino-based knock-down experiments for each of the Rh glycoproteins induced a significantly decreased ammonium excretion and subsequent ammonium accumulation in whole body lysates, confirming their crucial role for ammonium excretion after hatching until adulthood [35]. Since zebrafish larvae lack a functional urea cycle after hatching, they endogenously mimic the metabolic situation of patients with UCDs who suffer from the functional consequences of defective ureagenesis. Moreover, increasing the concentration of NH_4_^+^ ions in the water by addition of NH_4_Ac is a convenient way to induce acute hyperammonemia in zebrafish larvae [38]. This approach enabled us to investigate the effects of toxic ammonium concentrations on biochemical and gene regulation as well as survival in a per se urea cycle-deficient organism including the efficacy of different therapeutic interventions.

The pathophysiological basis of NH_4_^+^ toxicity to the brain was shown to be caused by NH_4_^+^-induced alterations in several metabolic pathways. Mice deficient for the urea cycle enzyme OTC exhibit decreased ATP and creatine content in brain as sign of cerebral energy depletion [39, 40]. Analogously, primary astrocyte cultures develop mitochondrial permeability transition upon NH_4_^+^ exposure resulting in cessation of ATP synthesis, production of reactive oxygen species and cell death [41, 42]. In addition, hyperammonemia increases osmotically active cerebral glutamine concentrations via astrocytic glutamine synthetase activity, which ultimately results in cytotoxic brain edema by astrocyte swelling [43, 44], a key finding that could also be verified in patients with UCDs by *in vivo* magnetic resonance spectroscopy [45]. Consistently, we observed a strong increase of glutamine in zebrafish exposed to NH_4_Ac at 4 dpf which was associated with gene induction of the metabolizing isoforms of glutamine synthetases, *Glula* and *Glulb*. In contrast to humans, the CNS of the zebrafish is characterized by the presence of radial glia instead of stellate or round astrocytes, which to some degree exert astrocyte-like functions. Mammalian astrocytes are functionally involved in neuronal injury response, the maintenance of ionic and osmotic balance and integrity of the blood-brain-barrier as well as the regulation of blood flow and synapse function [46–49]. Zebrafish radial glia are known to express the water transport-protein aquaporin-4 and additional markers of tight junctions, which are distinctive for human astrocytes [50, 51]. In light of glutamate transporter *Eaat2b* expression that colocalizes with *Glula* expression in the zebrafish CNS [52], the adaptive increase of glutamine concentrations together with gene induction of glutamine synthetases and glutaminases upon NH_4_^+^ exposure indicates that zebrafish larvae possess a metabolically active cerebral glutamine-glutamate-cycle. Strikingly, hyperammonemia caused a massive elevation of glutamate concentrations associated with depletion of ornithine and 2-oxoglutarate, pointing at transamination processes via OAT as important pathophysiological basis of NH_4_^+^-induced toxicity. Cataplerotic withdrawal of 2-oxoglutarate from the tricarboxylic acid (TCA) cycle due to glutamate synthesis has been proposed as a relevant factor for the observed energy depletion in hyperammonemia [53]. Increased pyruvate and lactate concentrations in the exposed zebrafish cohort again indicated that ATP is generated to a higher extent via anaerobic glycolysis suggesting impairment of TCA cycle flux. Increased lactate concentrations have also been observed in a rat model of acute liver failure as well as in primary astrocytes exposed to NH_4_^+^ [54]. Finally, HE is associated with decreased oxygen consumption *in vivo* [55, 56].

Glutamate is the major excitatory neurotransmitter of the mammalian brain by activating either ionotropic NMDA and α-amino-3-hydroxy-5-methylisoxazole-4-propionic acid (AMPA) receptors or metabotropic glutamate (mGlu1 to mGlu8) receptors [57, 58]. Excess production of glutamate at the synapse or inhibition of its reuptake from the synaptic cleft leads to an excessive stimulation of glutamate receptors, most importantly NMDA receptors, and consecutive calcium overload, shortage of ATP, mitochondrial dysfunction, increased oxidative stress resulting in excitotoxicity and cell death of adjacent neurons [59–63]. Thus, for proper neuronal functioning and prevention of neurotoxicity, it is essential for the organism to maintain extracellular cerebral glutamate concentrations within the normal range [64, 65]. Considerable evidence has been gathered demonstrating glutamate-induced neurotoxicity to be also a prominent pathophysiological factor in neurodegenerative diseases such as Huntington disease [66], Parkinson disease [67, 68] and amyotrophic lateral sclerosis [69, 70] as well as in acute insults to the brain including cerebral ischemia [71], perinatal brain injury [72] and epilepsy [73]. Our data indicate that excessive transamination-dependent glutamate production and cerebral glutamate accumulation with consecutive NMDA receptor-mediated neurotoxicity is a central pathophysiological hallmark of acute hyperammonemic conditions. Accordingly, *in vitro* NH_4_^+^ exposure of primary astrocytes and neuronal cultures induces glutamate release from astrocytes, inhibition of glutamate reuptake by astrocytes and excessive depolarization of glutamatergic neurons [74, 75]. Moreover, it has been shown that hyperammonemia is associated with increased synaptic glutamate release and an increased net formation of glutamate in different animal models *in vivo* [76–78], clearly supporting our findings. The selective downregulation of NMDA receptor subunits *Grin1a*, *Grin1b* and *Grin2da* observed in NH_4_^+^-exposed zebrafish larvae at 4 dpf most likely accounts for a compensatory mechanism to reduce the toxic effects of elevated cerebral glutamate concentrations by downsizing NMDA receptor-mediated signaling. As GRIN1a and GRIN1b are the subunits forming the Ca^2+^ channel of the NMDA receptor complex, their selective downregulation might represent an efficient adaptation process to suppress downstream events (e.g. alterations affecting NO metabolism, ATP shortage, mitochondrial dysfunction) induced by increased Ca^2+^ influx. A similar process involving decreased NMDA receptor density has been reported in rat and mouse models of acute and chronic hyperammonemia [79, 80]. This compensatory mechanism, however, fails at acute exposure to high ammonium concentrations like in our study.

Current therapeutic strategies in hyperammonemic decompensation of patients with UCDs are mainly based on nitrogen scavenging by intravenous administration of sodium benzoate and sodium or glycerol phenylbutyrate, forming water-soluble and dialyzable benzoylglycine (hippuric acid) and phenylacetylglutamine. These nitrogen scavengers are effective in lowering the concentration of ammonia by binding glycine and glutamine in the periphery, but do not directly interfere with secondary metabolic alterations in the brain (e.g. glutamatergic excitotoxicity and bioenergetic impairment), which ultimately result in brain damage and death.

Modulation of impaired glutamatergic neurotransmission has already been proposed as therapeutic concept in neurological diseases. Translational activators of glutamate transporter 1 (GLT1), such as ceftriaxone and LDN/OSU-0212320 have significant protective effects in animal models of amyotrophic lateral sclerosis and epilepsy [81, 82]. For hyperammonemic conditions, NMDA receptor antagonists MK-801 and 2-amino-5-phosphonovaleric acid improve neuronal survival in primary cortical neurons of newborn rats exposed to NH_4_^+^ [83]. NMDA receptor antagonists MK-801 and memantine have demonstrated neuroprotective properties in a rat model of acute liver failure increasing the percentage of surviving animals from 23% to 62%. However, this effect was restricted to animals suffering from mild acute liver failure [63]. Moreover, it has been shown that transient blockade of NMDA receptors induces apoptotic neurodegeneration in the developing brain of late fetal and neonatal rats [84]. Similarly, NMDA receptor blockade using the NMDA receptor antagonist memantine or ketamine slightly improved survival of NH_4_^+^-exposed zebrafish larvae in our study. Inhibition of glutamine synthetase to ultimately attenuate glutamine-mediated brain edema in acute hyperammonemia by application of L-MSO alone or in combination with memantine, however, did not further increase survival time proving that inhibition of cerebral glutamine synthetase has also a limited therapeutic efficacy for NH_4_^+^-exposed zebrafish.

Given the therapeutic limitations of glutamine synthetase inhibition and NMDA receptor blockade, we assumed that lowering brain glutamate concentrations by inhibition of OAT-mediated transamination could be an alternative approach to minimize cerebral glutamate excitotoxicity in acute hyperammonemia. Interestingly, a comparable approach involving transamination processes has already been used to attenuate glutamate excitotoxicity in a rat model of ischemic stroke. Administration of recombinant human aspartate aminotransferase to the animals was shown to lower serum and brain glutamate concentrations, which resulted in a significant reduction of stroke-induced infarct volume and sensorimotor deficit [85].

5-FMO is an irreversible, specific inhibitor of OAT competing with endogenous ornithine for the catalytic site of the enzyme [34]. *In vitro* determination of residual OAT activity after incubation with increasing concentrations of 5-FMO revealed pseudo-first-order inactivation kinetics with an apparent dissociation constant *K*_*i*_ of 70 μM. Mice injected intraperitoneally with 5-FMO in an effective concentration of 10 mg/kg body weight exhibited OAT inactivation to a maximum residual activity of 10 to 20%, which was resistant to further inactivation by higher or repeated doses [34]. Treatment of zebrafish larvae with 5-FMO not only normalized glutamate and consecutively glutamine concentrations, but also restored ornithine and arginine levels in a dose-dependent manner. Moreover, expression of NMDA receptor subunits *Grin1a* and *Grin1b* subsequently normalized upon treatment with 5-FMO, indicating efficient correction of altered glutamatergic neurotransmission in acute hyperammonemia. Adverse effects of treatment with 5-FMO were not observed. In our model system, therapeutic OAT inhibition was even by far superior to NMDA receptor blockade and inhibition of glutamine synthetase. More than 90% of exposed NH_4_Ac-exposed zebrafish survived an otherwise lethal dosage. This remarkable therapeutic efficiency owes to the position of OAT in intermediary metabolism linking 2-oxoglutarate metabolism and the TCA cycle to glutamatergic neurotransmission. As inhibition of OAT transamination activity in acute hyperammonemia leads to normalization of glutamate and glutamine levels consecutively restoring 2-oxoglutarate concentrations, it is a valuable therapeutic strategy to simultaneously tackle impaired glutamatergic neurotransmission, glutamine-mediated brain edema and cerebral energy depletion, which are the central culprits of hyperammonemia-induced neurotoxicity.

Therapeutic effects of OAT inhibition on reliable hard clinical endpoints such as brain damage and mortality in acute hyperammonemia have not been investigated in animal models before. Thus, our zebrafish model is exclusive in showing prevention of neurotoxicity and significantly prolonged survival times *in vivo*. Whether these promising beneficial effects of OAT inhibition on survival also hold true for mammalian organisms needs to be determined in future research. Given that inherited OAT deficiency is characterized by gyrate atrophy of the choroid and retina [86], long-term OAT inhibition as therapeutic strategy might bear the risk of inducing iatrogenic gyrate atrophy. Therefore, it would be desirable that new reversible inhibitors leading to a transient OAT inhibition will be developed for clinical use.

## Conclusion

Our finding that pharmacologic OAT inhibition prevents mortality in zebrafish exposed to lethal NH_4_^+^ concentrations opens new therapeutic avenues for individuals with UCDs, who are confronted with a lifetime risk of irreversible brain damage and death resulting from hyperglutaminergic and hyperglutamatergic hyperammonemia, a neurotoxic and often lethal biochemical triad.

## Supporting information

S1 Fig Kaplan-Meier plots of ammonium acetate-induced mortality at developmental stages 2 and 3 dpf. Zebrafish larvae (n = 100 per group) at 2 or 3 dpf were exposed to varying concentrations (5, 10 and 20 mM) of either NH_4_Ac or NaAc and survival rates monitored for up to 36 h. While zebrafish larvae did not succumb to NH_4_Ac in a dose range of 5 to 10 mM at either developmental stage (A, C), 20 mM NH_4_Ac induced death of 46% of exposed larvae at 3 dpf until the end of the observation period (log-rank test, P<0.001). Intriguingly, NH_4_-induced toxicity started not earlier than 24 h after start of exposure, equaling developmental stage 4 dpf of the exposed zebrafish cohort (C). NaAc had no toxic effect at either developmental stage (B, D).
(TIFF)

S2 Fig Negative control whole mount ISH of GLULA, GLULB and GLULC during zebrafish development. ISH was performed using sense-probes for the respective glutamine synthetase isoforms. Pictures are representative images of 3 independent experiments (n = 50 embryos/larvae per stage and experiment). Images show the embryonic heads at stages indicated. Left columns of each row show latter with anterior to the left and right columns with anterior to the top. Negative control ISH did not show any specific staining for each of the sense-probes used (A-C).
(TIF)

S3 Fig Relative mRNA expression of glutamine synthetases GLULA, GLULB and GLULC and glutaminases GLSA and GLS2B during zebrafish development. *Glula* showed a biphasic expression pattern with increased expression peaking at 75% epiboly followed by a consecutive decrease with a second peak appearing at 5 dpf (A). In contrast, *Glulb* and *Glulc* were both maternally delivered exhibiting decreasing expression during gastrulation (B, C). While *Glulc* remained hardly expressed during subsequent developmental stages (C), *Glulb* expression constantly increased after 24 hpf peaking at 5 dpf (B). *Glsa* expression constantly increased during neurulation with an expression peak at 5 dpf (D), whereas *Gls2b* displayed an exclusive expression peak at 5 dpf (E). Data are expressed as mean +/- SD in fold-change (whole body lysates, n = 3 with 50 larvae per group and experiment).
(TIFF)

S4 Fig Relative mRNA expression of transaminases GPT, GPT2 and OAT during zebrafish development. *Gpt* was expressed with only mild variation throughout whole embryonic development (A), whereas *Gpt2* exhibited highest expression levels during early gastrulation with a decrease at 75% epiboly (B). In contrast, *Oat* expression constantly increased during neurulation, peaking at 5 dpf (C). Data are expressed as mean +/- SD in fold-change (whole body lysates, n = 3 with 50 larvae per group and experiment).
(TIFF)

## Abbreviations

5-FMO: 5-fluoromethylornithine
ARG1: arginase 1
ASL: argininosuccinate lyase
ASS1: argininosuccinate synthetase 1
ATP: adenosine triphosphate
CNS: central nervous system
CPS1: carbamoylphosphate synthetase 1
dpf: days post fertilization
h: hours
HE: hyperammonemic encephalopathy
hpf: hours post fertilization
HPLC: high-performance liquid chromatography
ISH: in situ hybridization
M: mole
Min: minutes
mM: millimole
μM: micromole
NaAc: sodium acetate
NAGS: N-acetylglutamate synthase
NH_4_Ac: ammonium acetate
NMDA: N-methyl-D-aspartate
OAT: ornithine aminotransferase
OTC: ornithine transcarbamylase
sec: seconds
TCA: tricarboxylic acid
UCD: urea cycle disorder
