# Supplementary figures and images for: Pharmacologic rescue of hyperammonemia-induced toxicity in zebrafish by inhibition of ornithine aminotransferase
Source: PLoS One. 2018 Sep 10;13(9):e0203707. doi: 10.1371/journal.pone.0203707 (PMC6130883; doi:10.1371/journal.pone.0203707)

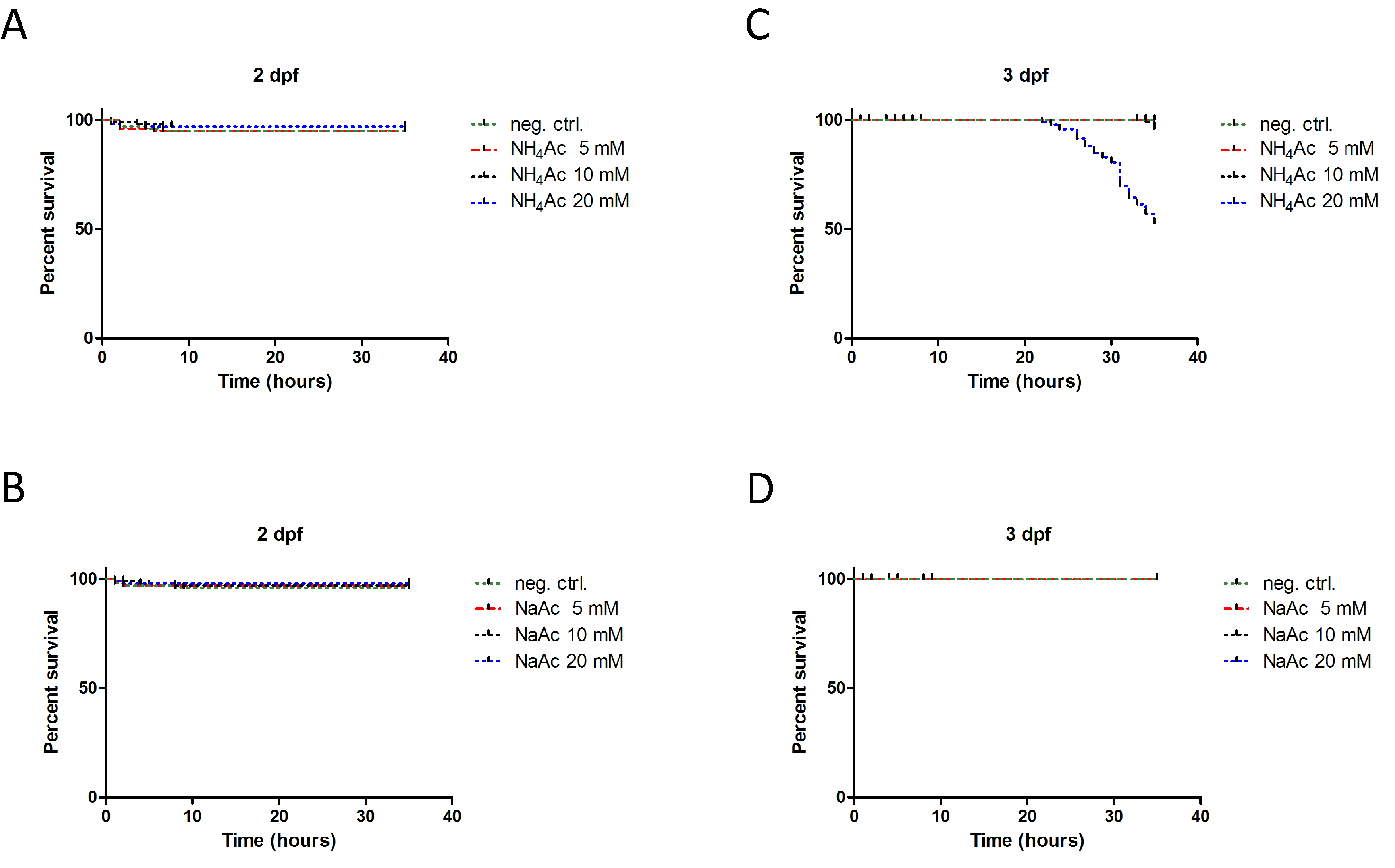

Supplement: S1 Fig — Zebrafish larvae (n = 100 per group) at 2 or 3 dpf were exposed to varying concentrations (5, 10 and 20 mM) of either NH4Ac or NaAc and survival rates monitored for up to 36 h. While zebrafish larvae did not succumb to NH4Ac in a dose range of 5 to 10 mM at either developmental stage (A, C), 20 mM NH4Ac induced death of 46% of exposed larvae at 3 dpf until the end of the observation period (log-rank test, P<0.001). Intriguingly, NH4-induced toxicity started not earlier than 24 h after start of exposure, equaling developmental stage 4 dpf of the exposed zebrafish cohort (C). NaAc had no toxic effect at either developmental stage (B, D). (TIFF) [file pone.0203707.s001.tiff]

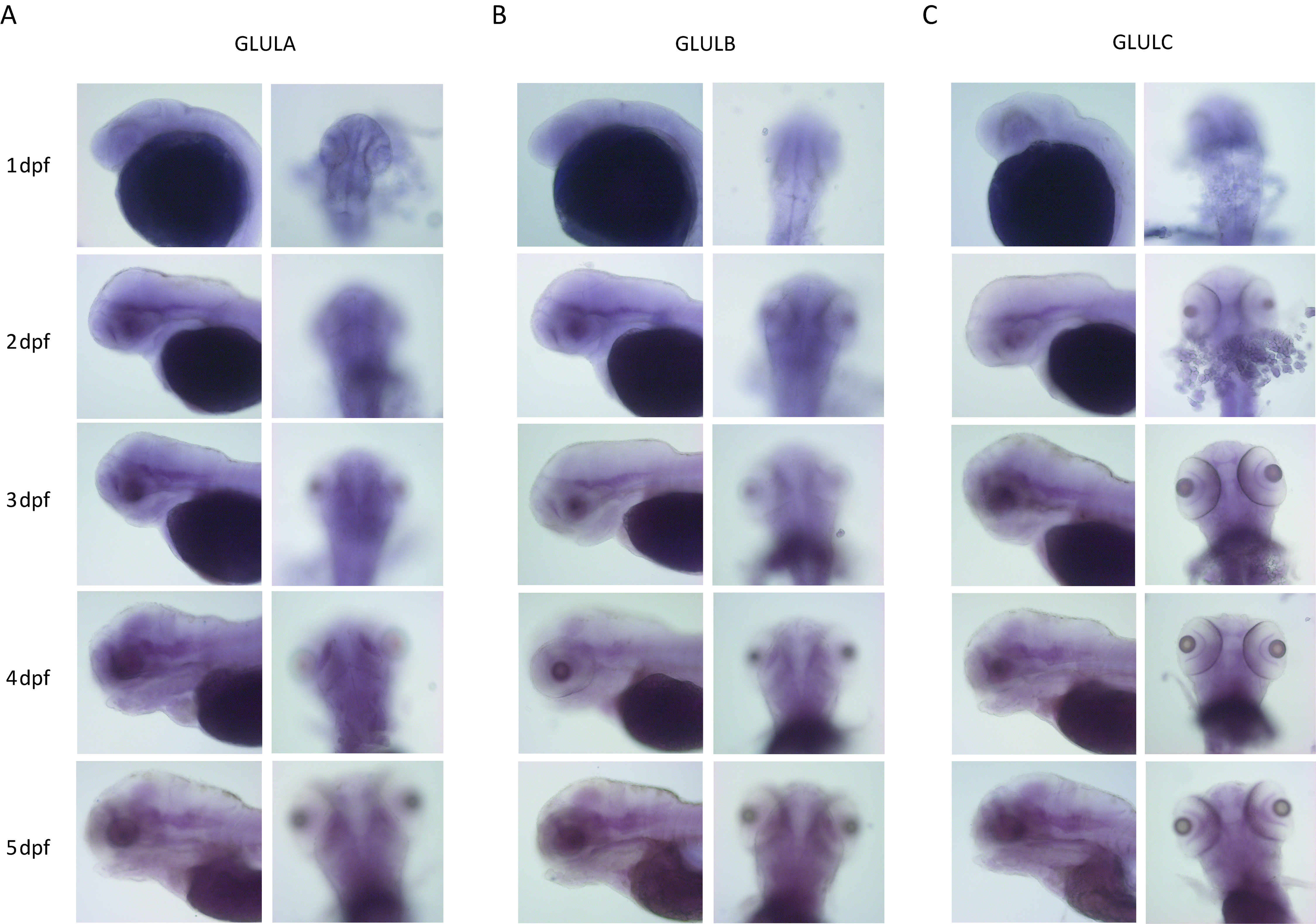

Supplement: S2 Fig — ISH was performed using sense-probes for the respective glutamine synthetase isoforms. Pictures are representative images of 3 independent experiments (n = 50 embryos/larvae per stage and experiment). Images show the embryonic heads at stages indicated. Left columns of each row show latter with anterior to the left and right columns with anterior to the top. Negative control ISH did not show any specific staining for each of the sense-probes used (A-C). (TIF) [file pone.0203707.s002.tif]

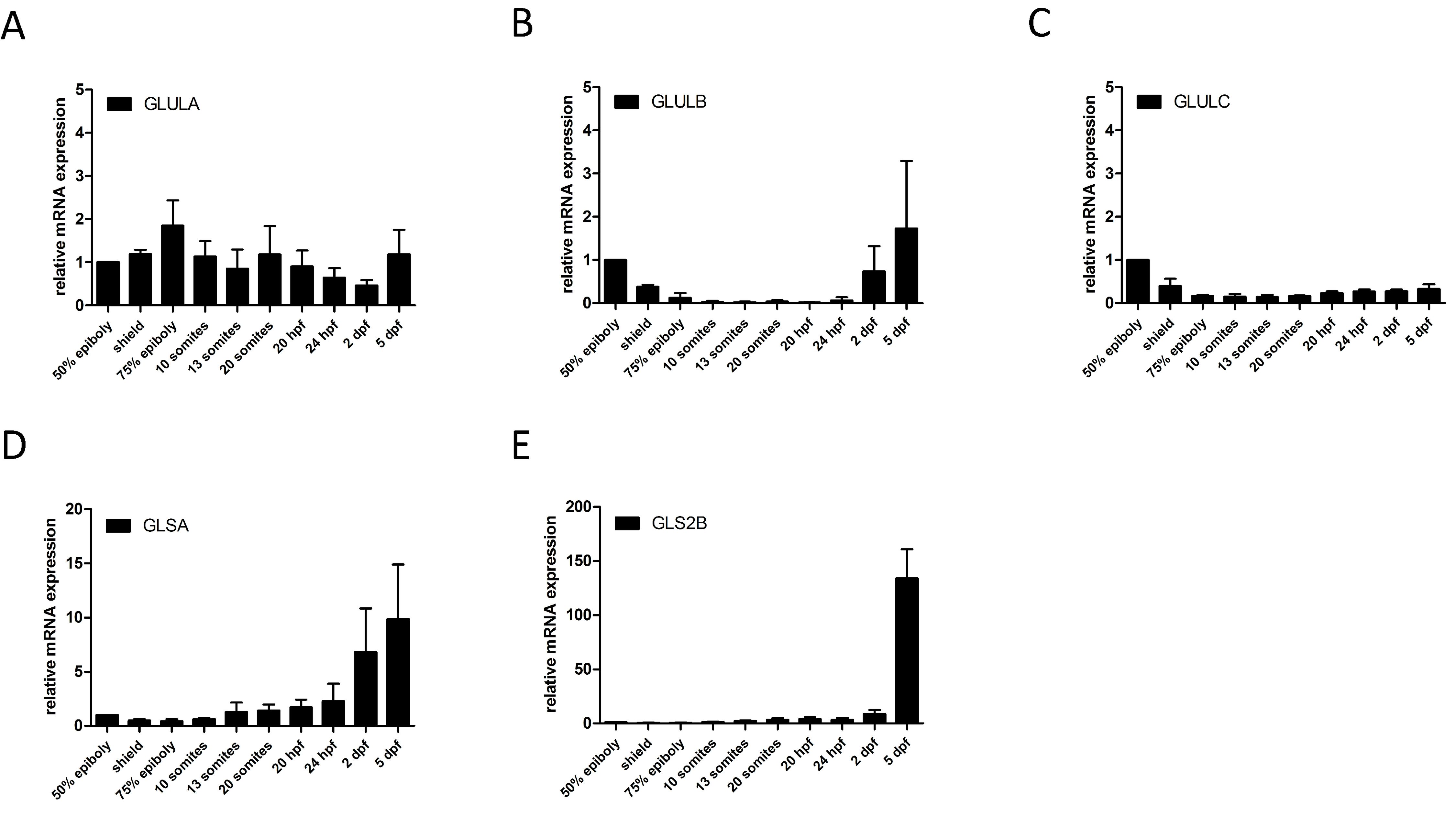

Supplement: S3 Fig — Glula showed a biphasic expression pattern with increased expression peaking at 75% epiboly followed by a consecutive decrease with a second peak appearing at 5 dpf (A). In contrast, Glulb and Glulc were both maternally delivered exhibiting decreasing expression during gastrulation (B, C). While Glulc remained hardly expressed during subsequent developmental stages (C), Glulb expression constantly increased after 24 hpf peaking at 5 dpf (B). Glsa expression constantly increased during neurulation with an expression peak at 5 dpf (D), whereas Gls2b displayed an exclusive expression peak at 5 dpf (E). Data are expressed as mean +/- SD in fold-change (whole body lysates, n = 3 with 50 larvae per group and experiment). (TIFF) [file pone.0203707.s003.tiff]

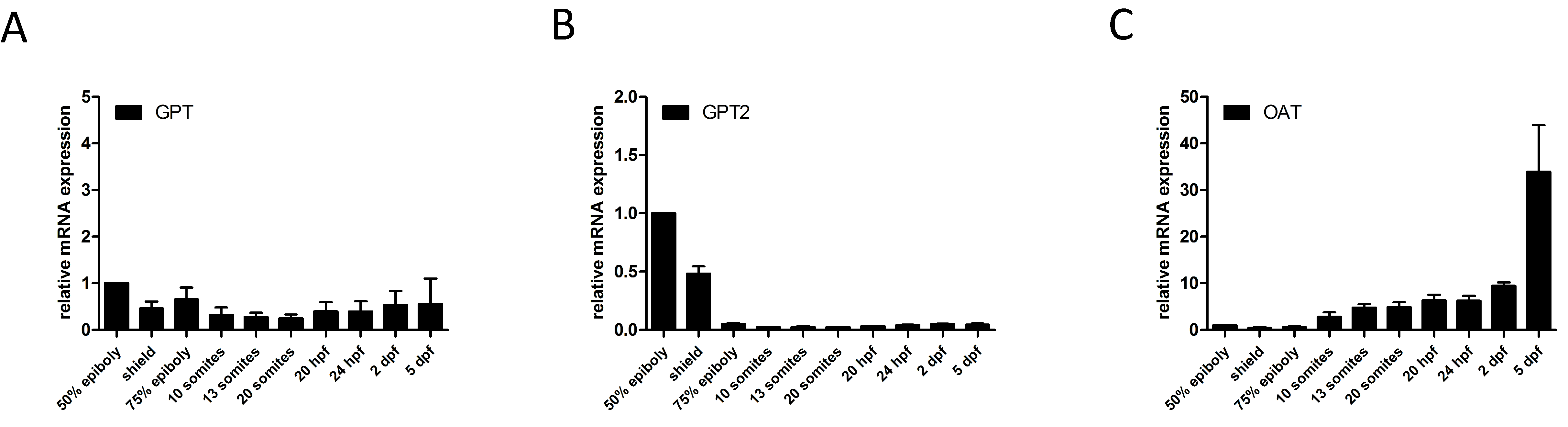

Supplement: S4 Fig — Gpt was expressed with only mild variation throughout whole embryonic development (A), whereas Gpt2 exhibited highest expression levels during early gastrulation with a decrease at 75% epiboly (B). In contrast, Oat expression constantly increased during neurulation, peaking at 5 dpf (C). Data are expressed as mean +/- SD in fold-change (whole body lysates, n = 3 with 50 larvae per group and experiment). (TIFF) [file pone.0203707.s004.tiff]
